# A cross-sectional matched sample study of nonsuicidal self-injury among young adults: support for interpersonal and intrapersonal factors, with implications for coping strategies

**DOI:** 10.1186/s13034-015-0070-7

**Published:** 2015-09-28

**Authors:** Heather C Trepal, Kelly L Wester, Erin Merchant

**Affiliations:** Department of Counseling, The University of Texas at San Antonio, 501 W. César E. Chávez Boulevard, San Antonio, TX 78207 USA; Department of Counseling and Educational Development, The University of North Carolina at Greensboro, PO Box 26170, Greensboro, NC 27402 USA

**Keywords:** Nonsuicidal self-injury, College students, Coping, Support

## Abstract

**Background:**

Young adults are a high-risk group for nonsuicidal self-injury (NSSI). It is important to have a better understanding of these behaviors in order to facilitate effective research, intervention, and treatment. Models have been presented to explain these behaviors where emotion regulation, coping, and support play a role. Yet conflicting results have occurred based on demographic factors such as race and sex. While controlling for the observable demographic factors, this study sought to examine differences between individuals who currently engage in NSSI, engaged in NSSI in the past, and never engaged in NSSI related to emotions, coping strategies, interpersonal support, and ethnic identity and belonging.

**Methods:**

Participants were selected from freshman students at two universities, in geographically different locations in the United States (*N* = 282). Participants in this study were matched on demographic factors: race, sex, and university. This led to demographically matched groups (current, past, never engagement in NSSI; *n* = 94 per group). Groups were compared on intrapersonal factors (i.e., emotions: depression and anxiety; coping strategies: adaptive and maladaptive; interpersonal support: family, friend, and significant other; and ethnic identity and belonging). Descriptive statistics and ANOVA with post hoc Scheffe were utilized to explicate differences between groups.

**Results:**

Individuals who never engaged in NSSI reported significantly higher levels of ethnic belonging and interpersonal support and lower levels of depression and anxiety than both groups who engaged in NSSI. Individuals who never self-injured used less adaptive and maladaptive coping strategies than participants who self-injured. Young adults who currently engaged in NSSI reported higher levels of depression and anxiety, higher levels of both types of coping, and perceived less support.

**Conclusions:**

It is important to understand the differences between individuals who self-injure in comparison to those who do not so that mental health clinicians can provide more effective services and preventative efforts.

## Background

A review of nonsuicidal self-injury (NSSI) reveals that, with the exception of inpatient populations, rates of NSSI are the highest among adolescents and young adults. More specifically, young adults in college students self-injure at a higher rate (up to 35%) [1] than the general population (1–6%) [2]. However, a recent review of longitudinal studies indicated that NSSI behaviors decrease by young adulthood [3]. Given that NSSI has been reported as one of the most difficult behaviors to treat [4], and that 80% of young adults who self-injure are not engaged in formal mental health treatment [5], there is a need to understand these behaviors in depth in order to facilitate effective clinical intervention and treatment.

Multiple models have been proposed to explain why individuals engage in NSSI, two of which highlight the need for emotion regulation to relieve distress [6, 7], and another which stresses the social and automatic functions of NSSI [8, 9]. Nock’s [7] Integrated Theoretical Model of the Development and Maintenance of NSSI maintains that the combination of intense aversive emotions and cognitions, with the added inability to cope or use of poor coping strategies, leads to engagement in NSSI to provide temporary regulation of the situation.

Empirical support has been found for these models. More specifically, it has been found that depression and anxiety are related to NSSI behaviors [10], and that self-injury is used to emotionally regulate these aversive emotions [9, 11–16]. In regards to specific coping strategies, individuals engaging in NSSI are significantly less likely to engage in problem or emotion focused coping, seek out instrumental support, or engage in religious or spiritual forms of coping; however, they are more likely to cope utilizing substance abuse, behavioral disengagement, and self-blame [5]. Interpersonal support from others has also been found to be important, with those who self-injure reporting less perceived support, communication, or belonging to family, peers, and significant others [17, 18].

Although there has been empirical support for these conceptual models of NSSI, it is difficult to distinguish these findings from the demographics (e.g., sex and race) of individuals who self-injure. For example, it was once assumed that females were the dominant group who self-injured [19]. Although no differences between females and males have been found in more recent research [15, 20, 21]. Problems with research design and analysis, such as lower samples sizes or a lack of statistical power (e.g., 19 males compared to 48 females) [22] may contribute to the lack of clarity related to sex differences in NSSI engagement. Another possible explanation may be that researchers have neglected examining gendered differences in NSSI behaviors due to ignoring specific methods that may be more likely utilized by males (e.g., hitting) [22].

In addition, White individuals have been found to have a higher prevalence of NSSI engagement than minority groups [20, 23]. Even so, researchers have been finding less of a difference in NSSI engagement between racial minorities and Whites [18] or opposite effects where minority groups, such as African Americans, report significantly higher rates of self-injury than Whites [23, 24]. Thus, there remains much more to be examined regarding the role of race and NSSI. To add another layer, ethnicity, particularly related to one’s sense of group belonging and affiliation appears to play a role. Wester and Trepal [15] found that individuals, regardless of race, who felt they belonged to their self-identified ethnic group, were less likely to engage in self-injury unless they were a member of the majority group at that institution. Thus, when individuals from minority racial groups attend a majority minority institution (e.g., a Hispanic student who attends a Hispanic Serving Institution), where they are a part of the dominant racial context, they are not less likely to self-injure. However, it does appear that ethnic group affiliation and belonging may provide a sense of support. Knowledge related to how race, ethnicity, and sex influence NSSI behaviors is extremely limited. More information is needed to better understand how these demographic factors play into NSSI engagement.

Demographic differences may also be confounding the actual relationship between NSSI and emotions, coping strategies and social support. For example, females have been reported to have higher levels of depression than males [25], which has been suggested to be due to selected coping strategies between men and women. More specifically, it has been found that males engage in physical and instrumental forms of coping, while females tend to ruminate, avoid, and be less active in their coping methods [26, 27]. This connects to what Wester and Trepal [5] found in regards to individuals who self-injure utilizing less instrumental, emotion, and problem focused coping strategies. Additionally, racial and ethnic differences have been found in regards to depression, anxiety, and coping strategies as well [28, 29].

The goal of the current study was to attempt to control for some of the observable demographic factors (e.g., race and sex) that have been found to influence NSSI behaviors, or that may cause group differences that are confounding with or independent of NSSI behaviors. Ho, Imai, King, and Stuart [30] suggested that engaging in a pre-matching process, where a database that can match individuals from one group (in this case NSSI engagement) to the control group (non-NSSI engagement), can bring the construct or variable “closer to being independent of background covariates which render any subsequent parametric adjustment either irrelevant or less important” (p. 200). They suggested that adjusting the data through matching for “potentially confounding control variables” prior to analysis can reduce the error and bias that can be found in raw data (p. 201).

Therefore the goal of the current study was to answer the following research question: While controlling for the observable demographic factors of sex and race that may have served as potential confounds in previous findings, what is the difference between individuals who currently engage in NSSI, engaged in NSSI in the past, and never engaged in NSSI related to emotions, coping strategies, interpersonal support, and ethnic identity and belonging?

## Methods

### Sample

The sample for the current study included 282 freshman students at two universities in the United States collected across two points in time (2008, 2011). This sample resulted from taking the freshman students from a larger sample (described below; *N* = 1,980) and first selecting the students who identified as currently engaging in nonsuicidal self-injury (NSSI). Currently engaging in NSSI was defined as self-reporting engaging in NSSI behaviors within the past 90 days and included a total of 99 participants in the sample. Once those who self-reported currently engaging in NSSI were identified, they were matched with students who had never self-injured and students who had previously self-injured (past NSSI) on race, sex, and university (the latter was matched given the two different locations). When more than one match existed for a currently engaged in NSSI participant, participants with complete data were randomly selected. Of the original 99 currently engaged in NSSI, five students could not be matched due to a lack of matching students in the other two self-injury categories, resulting in a total of 94 current engaged in NSSI students, 94 past engaged in NSSI students, and 94 never engaged in NSSI students (total *N* = 282) matched on race, sex, and university.

This sample of 282 originated from a larger sample of 1,980 college freshman. The matched participants (*N* = 282) did not significantly differ from the unmatched participants (*n* = 1,698) regarding age and sex. A significant difference did exist in regards to race (*X* = 21.01, *p* < 0.01). Specifically there were less Asians and African Americans, and significantly more Hispanic and Whites observed in the NSSI matched sample than expected. This is expected as researchers have found lower prevalence of self-harm behaviors among Asian and African American individuals [18]. Of the 282 freshmen student sample used in this study, the majority of participants were female (72%) with 28% identifying as male, with a mean age of 18.50 (*SD* = 2.32). The majority of participants were White (48.9%), followed by Hispanic (24.5%), Black/African American (10.6%), Multiracial (11.7%), and Asian (4.3%). Slightly over half of the sample came from University B (*n* = 150, 53.2%) with the remainder coming from University A (46.8%). Equal numbers from each sex, university, and racial category were present in current engagement in NSSI, past engagement in NSSI, and never engaged in NSSI groups as the groups were matched on these demographics.

### Procedures

Incoming freshman at the two universities (A and B) were targeted. University A was a midsized university located in the southeast United States and University B was a mid-sized Hispanic Serving Institution located in the southwest United State. Procedures from the two time points, and at both universities, were the same. Information for both the 2008 and 2011 samples will be provided here to better understand each individual sample; however, final sample demographics for the 282 participants were given in the sample section above for this study.

At both universities, freshman participants were randomly selected from the larger freshman student body. Specifically in 2008, a random selection of 2,400 incoming freshman consisted at University A and 8,000 at University B. Out of those freshmen, 1,396 students responded (13.5% response rate). Similarly, in 2011, a random selection of 2,525 freshmen from University A and 4,953 freshmen from University B was sampled. A total of 584 students responded (8% response rate: 300 University A; 284 University B). Samples were compared by data collection point, and by university, and no significant differences were found between groups on NSSI behavior variables or independent variables. Final respondents were similar to their university freshman student body on race, age, and sex. Therefore, the two universities and two time point samples were collapsed into one larger sample (*N* = 1,980), with the final matched sample being used for this study (*N* = 282).

Both the 2008 and 2011 freshman samples were sent an e-mail through their university email account inviting them to participate in this study. The e-mail contained a link to an online survey, which was the primary method of data collection. If they did not respond to the first e-mail they were sent a follow-up 1–2 weeks later for a total of three e-mails. Both samples had incentives for participation: In 2008 students were offered the possibility of winning one of three $50 raffles; in 2011 students were offered the possibility of winning an Apple iPod Touch.

### Instruments

Participants were asked to complete a demographic form that included sex, age, year in school, and race. They were also asked to complete measures of NSSI, ethnic identity, depression, anxiety, coping behaviors, and perceptions of interpersonal support. Each of these measures are described below.

#### Nonsuicidal self-injury

NSSI was measured through the use of an adapted version of the Deliberate Self Harm Inventory (ADSHI; original DSHI was developed by Gratz [1]). The ADSHI assessed NSSI engagement (yes/no), number and type of method, and frequency of engagement (count frequency within past 90 days). The ADSHI contains 12 items that assess for lifetime and current (90 days) engagement in particular NSSI behaviors (e.g., cut, burn, skin pick). If participants indicated they currently utilized a specific method of NSSI, they were asked to report their frequency of engagement with that method in the past 90 days. The ADSHI has been found to have adequate estimates of reliability (Cronbach α = 0.70 on both lifetime and current engagement [18, 31].

#### Ethnic identity

The Multi-Ethnic Identity Measure (MEIM) [32] was used to assess identification with participants’ self-identified ethnic group. The MEIM consists of 12 items rated on a 4-point Likert-type scale. The MEIM consists of two subscales [20]: Affirmation, Belonging, Commitment (MEIM-A) which measures the participant’s attitudes and feelings surrounding their identification with their ethnic group as well as the degree to which the participant identifies with their ethnic group; and Ethnic Identity Achievement (MEIM-EI), which measures the level of the participants understanding of and awareness about their ethnicity. Internal consistency of the measures for the current study was adequate (Cronbach α = 0.90 entire scale; MEIM-A α = 0.86; MEIM-EI α = 0.76).

#### Depression

The Center for Epidemiological Studies for Depression Scale (CES-D), short version [33] was used to measure participants’ level of depression. This assessment consists of a 10-item scale measuring the participant’s amount of depressive symptoms. Research has shown that the CES-D has good predictive accuracy for depression and adequate reliability (0.64), with Cronbach’s α of 0.61 in the current study. High scores on this assessment indicate higher levels of depression.

#### Anxiety

The 5-item Anxiety subscale of the PGI General Well Being Scale [34] was used to assess for anxiety. Respondents answered each item on a 4-point Likert-type scale, rating the frequency of occurrence of each item. High scores on this subscale indicate higher levels of wellness and less anxiety. Cronbach’s alpha for the current study was .78.

#### Coping

The Brief COPE [35], which consists of 28-item to assess 14 different coping styles, was used to assess maladaptive and adaptive coping. Participants rate their use of various coping skills from (0) “I usually don’t do this at all” to (3) “I usually do this a lot”. This assessment has scale reliabilities of 0.71 [35]. For the purpose of this study the various coping styles were organized into two subscales. The first subscale is Avoidant/Maladaptive Coping (Cronbach’s alpha = 0.73), which consists of denial, self-distraction, venting, substance abuse, behavioral disengagement, and self blame. The second subscale is Active/Adaptive Coping (Cronbach’s alpha = 0.79), which consists of active, planning, instrumental support, positive reframe, humor, acceptance, religion, and emotional support.

#### Interpersonal support

The Multidimensional Scale of Perceived Social Support (MSSPSS) [36] utilizes a Likert scale (“very strongly” to “disagree”) to assess for an individual’s perceived social support from family, friends, and significant others. For this study, the full scale of interpersonal support was used, in addition to the three individual subscales. In previous studies reliability for this assessment has been found to range on the scales from .81 to 0.98. Cronbach alpha in the current study were 0.91.

### Data analysis

Descriptive statistics were conducted to examine the NSSI behaviors engaged in by the current and past NSSI groups. One-way ANOVAs were used to examine if the current, past, and never engaged in NSSI groups significantly differed on coping, interpersonal support, depression, anxiety, and ethnic identity after they were matched on sex, race, and university (environmental context). Due to the matched nature of this sample, if one of the participants was missing data on a particular scale (e.g., depression), all matched individuals were removed from that analysis.

## Results

Ninety-four individuals indicated that they currently engaged in NSSI behaviors within the past 90 days of completing the survey. For these individuals, the average number of methods they used throughout their life was 2.56 (*SD* = 1.81, mode = 1.00), with the current average number of methods used in the past 90 days being 2.09 (*SD* = 1.97, mode = 1.00). The frequency of engagement, or number of episodes, participants reported in the past 90 days ranged from 1 to 1,000 (*M* = 24.46, *SD* = 112.90; note: one person who reported engaging over 5,000 time in the past 90 days was removed from this mean score due to the outlying score; 13 individuals indicated the methods they currently utilized to NSSI but did not report a frequency). Individuals who reported engaging in past NSSI, but not within the past 90 days, reported having utilized an average of 1.55 methods (*SD* = 1.06).

### Emotions: depression and anxiety

NSSI groups were significantly different on levels of depression and anxiety (*F* (2, 266) = 69.56, *p* < 0.001, *η*^2^ = 0.35; *F* (2, 275) = 19.88, *p* < 0.001, *η*^2^ = 0.13, respectively). Post hoc Scheffé was used to determine which specific groups significantly differed. For depression, individuals who never engaged in NSSI reported significantly lower levels of depression than those with past engagement and current engagement in NSSI (see Table 1). Additionally, individuals who engaged in NSSI in the past reported significantly lower levels of depression than individuals currently engaging in NSSI. Similarly for anxiety, individuals who never engaged in NSSI reported significantly lower levels of anxiety than individuals who engaged in NSSI in the past or currently; however, no significant differences existed in levels of reported anxiety between past and current engagement in NSSI groups.

**Table 1:** Differences between Never, Past, and Current NSSI Engagement groups on emotions, coping, and interpersonal supports

	Current NSSI				Past NSSI				Never NSSI				η^2^
	*M*	SD	95% CI		*M*	SD	95% CI		*M*	SD	95% CI		
			*LL*	*UL*			*LL*	*UL*			*LL*	*UL*	
Emotions													
Depression (*n* = 89/group)*	19.73^a^	6.67	18.32	21.13	15.10^b^	7.66	13.49	16.72	8.48^c^	4.41	7.55	9.41	0.34
Anxiety (*n* = 92/group)	13.03^a^	3.21	12.39	13.69	13.86^a^	2.78	13.28	14.41	15.63^b^	2.54	15.09	16.13	0.13
Coping													
Adaptive (*n* = 90/group)	38.23^a^	11.86	35.79	40.66	36.29^a^	11.83	33.61	38.57	33.47^b^	8.05	31.78	35.15	0.03
Maladaptive (*n* = 90/group)	22.49^a^	7.78	20.80	24.04	17.22^b^	7.78	15.52	18.76	11.82^c^	6.26	10.51	13.13	0.26
Interpersonal support													
Family support (*n* = 93/group)	20.28^a^	6.82	18.88	21.68	22.01^b^	5.34	20.91	23.11	24.48^c^	4.37	23.58	25.38	0.09
Friend support (*n* = 93/group)	22.10^a^	5.78	20.93	23.26	23.25^a,b^	4.60	22.30	24.19	24.58^b^	4.22	23.71	25.45	0.04
Significant other support (*n* = 93/group)	21.44^a^	6.69	20.07	22.82	23.94^b^	5.50	22.80	25.07	24.57^b^	4.70	23.60	25.54	0.05
Ethnic identity (*n* = 93/group)	2.54	0.68	2.42	2.70	2.52	0.72	2.37	2.67	2.49	0.70	2.35	2.64	0.01
Belonging (*n* = 93/group)	2.38^a^	0.69	2.24	2.52	2.58^a^	0.79	2.42	2.75	3.09^b^	0.55	2.98	3.20	0.16

Different superscript letters (^a, b, c^) signify the group significantly differed.

* Sample size per group is noted by each dependent variable, as noted earlier if one person in the triad match was missing a scale score the entire matched triad was removed from the analysis to ensure matched demographic data remained constant. No one participant was missing data from all dependent variables.

### Coping: adaptive and maladaptive

**Fig. 1:**
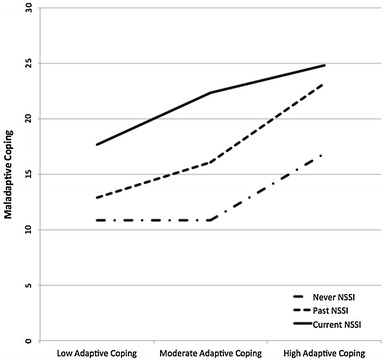
Degree of employing maladaptive coping strategies by adaptive coping strategies and NSSI engagement category.

Adaptive and maladaptive coping significantly differed by NSSI group (*F* (2, 269) = 4.49, *p* < 0.05, *η*^2^ = 0.03; *F* (2, 270) = 47.88, *p* < 0.001, *η*^2^ = 0.26, respectively). Individuals who never engaged in NSSI and those currently engaging in NSSI significantly differed, with individuals currently engaging employing greater levels of adaptive coping strategies. However, individuals who engaged in NSSI in the past did not significantly differ from those who currently engaged in NSSI or those who never engaged in NSSI. Similarly to adaptive coping, individuals who currently engaged in NSSI also reported employing greater amounts of maladaptive coping skills than those who never engaged in NSSI, however they also used greater amounts of maladaptive coping than individuals who reported past NSSI engagement. Additionally, those who engaged in NSSI in the past reported significantly higher levels of maladaptive coping than those who never engaged in NSSI. To better understand the connection between high levels of maladaptive and adaptive coping strategies employed by individuals who currently engaged in NSSI compared to the other two groups, they were graphed by taking the top, middle two, and lower quartiles of adaptive coping and graphing them with maladaptive coping scores for each NSSI group (see Fig. 1). As can be seen, regardless of high or low levels of utilizing adaptive coping strategies, those who currently engaged in NSSI also reported greater levels of maladaptive coping, followed by those who engaged in NSSI in the past, with the lowest level of maladaptive coping employed by the never engaged in NSSI group in all low, moderate and high adaptive coping quartiles. Interestingly, all individuals regardless of group who employed high levels of adaptive coping also engaged in greater use of maladaptive strategies as well.

To further examine how adaptive and maladaptive coping differed by NSSI engagement, a follow-up ANOVA analysis on the specific coping skills was conducted (see Table 2). Focusing on maladaptive forms of coping, all six maladaptive coping strategies were found to significantly differ. Individuals currently engaging in NSSI reported utilizing all six forms of maladaptive coping significantly more than individuals who never self-injured. Similarly, those who currently engaged in NSSI reported utilizing five of the six maladaptive coping methods at greater levels than those who engaged in NSSI in the past. The only coping strategy these two groups did not significantly differ on was venting. Finally, individuals who reported engaging in NSSI in the past reported higher employment of venting, substance abuse, behavioral disengagement, and self-blame strategies than those who never engaged in NSSI; however, these two groups did not differ on the degree to which they used self-distraction and denial forms of coping.

**Table 2:** Differences across Never, Past, and Current NSSI groups on specific coping strategies employed

	Current NSSI				Past NSSI				Never NSSI				η^2^
	*M*	SD	95% CI		*M*	SD	95% CI		*M*	SD	95% CI		
			*LL*	*UL*			*LL*	*UL*			*LL*	*UL*	
Adaptive emotions													
Active coping	5.14^a^	1.76	4.78	5.51	4.91^a,b^	1.66	4.57	5.26	4.51^b^	1.38	4.22	4.79	0.03
Planning coping	5.28^a^	1.82	4.91	5.66	4.96^a,b^	1.77	4.59	5.32	4.65^b^	1.38	4.36	4.94	0.02
Positive Reframe	4.97	2.09	4.53	5.40	4.43	1.96	4.03	4.84	4.32	1.55	4.00	4.64	0.02
Acceptance	5.76^a^	1.81	5.39	6.14	5.11^b^	1.76	4.74	5.48	4.76^b^	1.40	4.47	3.78	0.06
Humor	4.40^a^	2.38	3.91	4.89	4.15^a,b^	2.08	3.72	4.58	3.42^b^	1.69	3.08	3.78	0.04
Religion	3.51	2.50	2.99	4.03	3.26	2.51	2.74	3.79	3.53	2.10	3.09	3.97	0.00
Seek emotional support	4.49	2.14	4.05	4.93	4.66	1.94	4.26	5.06	4.16	1.65	3.81	3.97	0.01
Seek instrumental support	4.67	2.11	4.23	5.11	4.54	1.90	4.15	4.94	4.07	1.68	3.72	4.42	0.02
Maladaptive coping													
Denial	2.45^a^	1.85	2.07	2.83	1.52^b^	1.47	1.21	1.82	1.09^b^	1.53	0.77	1.41	0.11
Self-distraction	5.58^a^	1.81	5.20	5.96	4.79^b^	1.77	4.42	5.16	4.29^b^	1.32	4.01	4.56	0.10
Venting	3.56^a^	1.69	3.60	4.31	3.43^a^	1.83	3.05	3.81	2.52^b^	1.60	2.19	2.86	0.11
Substance use	2.43^a^	1.87	2.04	2.82	1.41^b^	1.53	1.09	1.72	.52^c^	1.39	.23	.81	0.19
Behavioral disengagement	3.03^a^	1.88	2.64	3.42	2.13^b^	1.75	1.77	2.50	1.19^c^	1.54	.87	1.51	0.16
Self-blame	4.97^a^	2.04	4.54	5.39	3.87^b^	2.17	3.42	4.32	2.21^c^	1.80	1.84	2.59	0.24

Different superscript letters (^a, b, c^) signify the group significantly differed.

Examining adaptive forms of coping, significant differences were found between the three NSSI groups on five of the eight strategies. No significant difference was found between groups on engaging in religious coping strategies, seeking out emotional support or instrumental support. Significant differences were found on the amount to which active coping, planning strategies, positive reframing, acceptance, and humor was used to cope. In post hoc Scheffe analyses, no significant differences were found between the three groups on positive reframing. Similar to maladaptive coping strategies, individuals who reported currently engaging in NSSI reported higher use of active coping, planning, acceptance, and humor strategies than individuals who never engaged in NSSI. However, past and current engaged NSSI groups did not significantly differ in their use of any adaptive coping strategy, with the exception of acceptance strategies. Additionally, past and never engaged in NSSI groups did not significantly differ on any adaptive coping strategies.

### Perceived interpersonal support

The perceived support felt from family, friends, and significant others was explored across NSSI engagement groups. A significant difference was found between groups on each of the three forms of perceived support (*F* (2, 279) = 13.25, *p* < 0.001, *η*^2^ = 0.08 for family; *F* (2, 277) = 6.09, *p* < 0.01, *η*^2^ = 0.04 for friend; *F* (2, 279) = 7.87, *p* < 0.001, *η*^2^ = 0.05 for significant other). With all three forms of interpersonal support, individuals who never engaged in NSSI reported significantly higher perceived levels of support than individuals currently engaging in NSSI. However, the never engaged in NSSI group did not significantly differ on perceived friend or significant other support than those who engaged in NSSI in the past; yet these two groups did significantly differ on the perception of support from family. Individuals currently engaging in NSSI reported similar levels of support from family and friends as those who engaged in the past, but reported significantly lower levels of support from significant others than individuals who engaged in NSSI in the past.

### Ethnic identity and sense of belonging

Significant differences were found for the ethnic belonging scale (*F* (2, 278) = 26.58, *p* < 0.001, *η*^2^ = 0.16) but not for the ethnic identity scale (*F* (2, 278) = 0.13, *p* > 0.05, *η*^2^ = 0.001). Specifically for ethnic belonging, individuals who never engaged in NSSI reported significantly higher levels of ethnic belonging than the past and current NSSI engagement groups; however, the latter two groups did not significantly differ.

## Discussion

This study is one of the first to employ a matched sample of engagement in NSSI (current, past, and never) in an attempt to control confounding variables between the samples that may result in differences between those who self-injure and those who do not. Results that compared interpersonal and intrapersonal factors, such as emotions, coping, interpersonal support and ethnic identity and sense of belonging, indicted that the three groups differed in significant ways.

The never engaged in NSSI group reported the lowest levels of both depression and anxiety. In addition, those who currently engaged in NSSI reported more depression than the other groups. With regard to anxiety, both the current and past engagement in NSSI groups reported similar levels of anxiety. These findings are consistent with proposed models of NSSI engagement and support past research that there may be a connection between these emotions and NSSI [9–14, 16]. Additionally, Nock et al. [15] found that 85–90% of individuals engaged in NSSI to relieve emotions through automatic negative reinforcement functions. Chickering and Reisser [37] identified the college years as a time of learning to manage emotions. Given the results from this study, mental health clinicians should note that college students who currently engage in NSSI might be struggling with intense emotions such as depression and anxiety and need tools to learn how to effectively manage them.

One way that college students attempt to manage intense emotions is by employing coping strategies. In this study, there were also differences in all three groups regarding coping. For example, those who currently engage in NSSI reported using more adaptive and maladaptive coping strategies than either of the other two groups. This finding makes sense given the increased amounts of depression and anxiety they reported. They might need to utilize a greater amount of coping than individuals who are not suffering from high levels of depression and anxiety. Nock et al. [15] found that youth who self-injured were able to delay engagement in NSSI by using alternative coping strategies such as distraction or talking to someone. However, the difference in this study is that these young adults actually engaged in NSSI, and were still employing greater numbers of both adaptive and maladaptive coping strategies. Specifically, those who currently engaged in NSSI reported employing all 6 types of maladaptive coping (i.e., denial, self-distraction, venting, substance abuse, behavioral disengagement, self blame) more than those who never engaged in NSSI and using 5 of the 6 strategies more than those who engaged in NSSI in the past. This suggests a few possibilities, one of which highlights the low distress tolerance of individuals who engage in NSSI thus revealing a greater need to use multiple coping methods [6, 9], as well as the possibility that the coping strategies employed may not be alleviating the aversive emotions of depression and anxiety. Thus, the need to continue employing more and more coping methods, potentially not doing so effectively, and potentially resulting in engagement in NSSI.

Wester and Trepal [5] previously determined that the ability to adaptively cope was negatively related to engaging in NSSI. As stated earlier, adaptive coping (i.e., active, planning, instrumental support, positive reframe, humor, acceptance, religion, and emotional support) also differed between groups. However, in this study, individuals who currently engaged in NSSI behaviors actually used more adaptive coping strategies. Therefore, they used greater numbers of both maladaptive methods than the two others groups, and greater numbers of adaptive methods than the never engaged in NSSI group but equal amounts as the past NSSI engagement group. Interestingly, all individuals regardless of group who employed high levels of adaptive coping also engaged in greater employment of maladaptive strategies as well. Chickering and Reisser’s [37] assertion that college is a developmental time of learning to manage emotions can explain this to some degree; specifically that overwhelming emotions have the power to derail the educational process for young adults. College students are faced with new situations and may experiment with both types of coping strategies as a result. However, this need to manage emotions does not completely explain the higher levels of both strategies for those currently engaged in NSSI, other than they have higher levels of depression and anxiety. What is left unanswered is whether the individuals in this study were using these high levels of coping strategies to delay or not engage in NSSI, and yet still ultimately they still engaged. Therefore, would engagement have been higher without these maladaptive and adaptive coping strategies? More research needs to be conducted to determine if these strategies delay or help individuals avoid engaging in NSSI, or if the use of these strategies is not effective, thus the coping behaviors being employed are being done so inadequately.

While coping strategies differed among groups, it was also found that individuals who never self-injured reported a higher level of support from friends, family and significant others than those who currently engaged in NSSI. Finding ways to cultivate various types of supportive relationships may be an important protective factor for those who self-injure [17, 18]. Whisenhunt et al. [38] and Buser et al. [39] determined that social support was necessary in decreasing or extinguishing NSSI behavior. In particular, the results of this current study indicated that those who currently self-injure perceived less support from family and significant others than both other groups, and less peer support than those who never self-injured. This may be due to criticism felt from family prior to engaging in NSSI [40] or reactions of family once they were aware of the self-injury [41].

Similar to a sense of interpersonal support, the only group who significantly endorsed ethnic belonging was the never engaged in NSSI group, leaving those who engaged in NSSI, currently or in the past, indicating they felt less association and belonging to their self-identified ethnic group. This may be an important finding as higher levels of ethnic belonging have been identified as a protective factor against NSSI [18]. Mental health professionals should take notice of this finding and look for ways to assess, enhance, and encourage ethnic belonging with children and adolescents and their families, as this may be a protective factor against depression, as well as NSSI. Researchers may want to further investigate the specific role of ethnic belonging relative to NSSI.

Finally, researchers should also take note of the matched sample approach (as recommended by Ho, Imai, King, & Stuart [30]) when conducting future studies with those who self-injure. The advantage of this type of matching on demographic categories (e.g., race and sex) allows for the minimization of potentially confounding variables when examining NSSI.

### Limitations

Although the results of this study have both research and clinical implications, limitations do exist. For example, there was a low participation rate of 8–13.5% of the university freshmen from both campuses, thus reducing the value of the main findings. In addition, the majority of the sample was predominantly female (72%) and White (48.9%) and Hispanic (24.5%), and this was perhaps more reflective of the universities these participants attended and not of the college student population as a whole. A more demographically diversified sample may have produced different results.

### Clinical implications

The findings in the current study provide mental health clinicians some concrete ways to intervene. Even though various evidence-based practices exist (e.g., DBT, problem solving therapy, CBT), it still remains that clinicians have indicated clients who self-injure are the most difficult to treat [4]. Thus regardless of the therapeutic method or intervention a clinician is using with a client who engages in self-injury, it is imperative that they inquire not only about emotive symptoms but also about social support, sense of belonging, and coping strategies. While assisting individuals in reaching out, communicating, and developing relationships with others, it is suggested, due to the findings in this study, that while clinicians may inquire about alternative coping methods used instead of NSSI, that they also need to explore how these coping methods are being implemented and how effective they actually are for the individual client. Thus, are these methods being used truly delaying engagement in NSSI behaviors? If not, the counselor may actually need to walk the client through how to implement various coping strategies, instead of assuming that the client knows because they identify a list of various strategies they utilize. Future studies may investigate the role of counseling, NSSI, and coping specifically exploring how these strategies are used by clients who self-injure and the role in which engagement in counseling may play.

## Conclusion

Given that college students’ self-injure at high rates [1] it is important for researchers to continue to investigate explanatory models of these behaviors. This study, which investigated components of several NSSI models [6, 7], found that there are important differences between those who currently engage in NSSI and those who have never engaged in NSSI and who engaged in NSSI in the past while controlling for various demographic factors. Specifically, those who currently engaged in NSSI are more likely to be experiencing depression and anxiety, employ more adaptive and maladaptive coping methods, and perceive less support. Mental health clinicians are encouraged to note these differences when engaging college students who NSSI in treatment.
